# Aspirin use and risk of breast cancer in African American women

**DOI:** 10.1186/s13058-020-01335-1

**Published:** 2020-09-04

**Authors:** Kimberly A. Bertrand, Traci N. Bethea, Hanna Gerlovin, Patricia F. Coogan, Lauren Barber, Lynn Rosenberg, Julie R. Palmer

**Affiliations:** 1 Slone Epidemiology Center at Boston University, 72 East Concord Street, L-7, Boston, MA 02118 USA; 2 Georgetown Lombardi Comprehensive Cancer Center, Washington, DC, USA; 3 Massachusetts Veterans Epidemiology and Research Information Center, Veterans Affairs Boston Healthcare System, Boston, MA USA; 4 Department of Epidemiology, Boston University School of Public Health, Boston, MA USA

**Keywords:** Aspirin, Non-steroidal anti-inflammatory drugs, Breast cancer, African American, Risk

## Abstract

**Background:**

Use of aspirin and other non-steroidal anti-inflammatory drugs (NSAIDs) has been hypothesized to be associated with reduced risk of breast cancer; however, results of epidemiological studies have been mixed. Few studies have investigated these associations among African American women.

**Methods:**

To assess the relation of aspirin use to risk of breast cancer in African American women, we conducted a prospective analysis within the Black Women’s Health Study, an ongoing nationwide cohort study of 59,000 African American women. On baseline and follow-up questionnaires, women reported regular use of aspirin (defined as use at least 3 days per week) and years of use. During follow-up from 1995 through 2017, 1919 invasive breast cancers occurred, including 1112 ER+, 569 ER−, and 284 triple-negative (TN) tumors. We used age-stratified Cox proportional hazards regression models to estimate hazard ratios (HRs) and 95% confidence intervals (CIs) for associations of aspirin use with risk of ER+, ER−, and TN breast cancer, adjusted for established breast cancer risk factors.

**Results:**

Overall, the HR for current regular use of aspirin relative to non-use was 0.92 (95% CI 0.81, 1.04). For ER+, ER−, and TN breast cancer, corresponding HRs were 0.98 (0.84, 1.15), 0.81 (0.64, 1.04), and 0.70 (0.49, 0.99), respectively.

**Conclusions:**

Our findings with regard to ER− and TN breast cancer are consistent with hypothesized inflammatory mechanisms of ER− and TN breast cancer, rather than hormone-dependent pathways. Aspirin may represent a potential opportunity for chemoprevention of ER− and TN breast cancer.

## Introduction

Relative to US white women, African American women have a disproportionately high incidence of aggressive breast cancer subtypes, such as estrogen receptor (ER)-negative tumors [1–3], and higher mortality from breast cancer [4]. Inflammation may play a role in breast cancer development, especially ER− breast cancer. Aspirin is an anti-inflammatory agent and a weak aromatase inhibitor [5, 6]. Experimental studies have demonstrated the chemopreventive properties of aspirin and other non-steroidal anti-inflammatory drugs (NSAIDs) [7]. In population-based studies, there is consistent and compelling evidence that regular use of aspirin is inversely associated with colorectal cancer incidence [8]. The use of aspirin may also reduce the risk of breast cancer; however, results from epidemiologic studies are inconsistent [9, 10].

Few studies have investigated these associations among African American women. A previous Black Women’s Health Study (BWHS) analysis of aspirin use and 12-year risk of breast cancer showed a statistically significant inverse association [11]; however, that analysis did not consider ER+ and ER− breast cancer separately, nor have any previous studies in African American women evaluated whether associations differ by ER subtype. The present analysis updates our previous report with an additional 10 years of follow-up and consideration of associations by ER status.

## Methods

### Study population

The BWHS is an ongoing prospective cohort of 59,000 African American women, who were ages 21 to 69 years (median age, 38 years) at baseline in 1995. Every 2 years, follow-up questionnaires are sent to participants to update information on incident cancer diagnoses and reproductive and behavioral factors, including medication use [12]. Notices of deaths are obtained from next-of-kin, the US Postal Service, and yearly searches of the National Death Index.

For this analysis, women were excluded if they had been diagnosed with breast cancer (*n* = 743) or any other type of cancer (except non-melanoma skin cancer) (*n* = 696) before baseline in 1995, or if they did not answer the question about aspirin use on the baseline questionnaire (*n* = 4399); the final analytic cohort included 53,162 African American women ages 21–69 at baseline. The study protocol was approved by the Boston University Institutional Review Board (IRB) and by the IRBs of participating cancer registries as required.

### Case ascertainment

Incident cases of breast cancer in the BWHS were ascertained through self-report on biennial follow-up questionnaires (95% of cases) or identified through death records or linkage to 24 cancer registries in states covering 95% of participants (5% of cases). Breast cancer diagnoses were confirmed by review of medical records, pathology reports, and cancer registry records. Data on tumor characteristics were also abstracted from these records.

Among 53,126 women in the BWHS who were free from cancer and had available information on aspirin use at baseline, there were 1919 invasive breast cancers diagnosed through 2017. Information on ER status was available for 88% of these cases (569 ER− and 1112 ER+ tumors). Of the 569 ER− breast cancers, HER2 status was known for 368 (65%); 284 (77%) of these were classified as triple-negative (TN) cases.

### Exposure and covariate assessment

Regular medication use, defined as use at least 3 days per week, was queried on baseline and biennial follow-up questionnaires. On the baseline questionnaire, women reported current use of aspirin or acetaminophen, separately, at least 3 days per week. Current users were also asked to report the duration of use (< 1, 1, 2, 3–4, ≥ 5 years). Popular brand names (e.g., Bayer, Anacin, Tylenol) were given as a prompt. Regular use of other NSAIDs (e.g., ibuprofen) at least 3 days per week was queried beginning in 2009. Prior to 2009, non-aspirin NSAID use was reported via open-text response and coded using the Slone Drug Dictionary [13]; however, the duration of use was not recorded for open-text responses. Identical questions on medication use were included on follow-up questionnaires. Information on dose was not obtained.

The baseline questionnaire collected information on established and putative risk factors for breast cancer including adult height, weight, age at menarche, oral contraceptive use, parity, age at first birth, lactation, menopausal status, postmenopausal hormone use, alcohol consumption, physical activity, and breast cancer in first-degree relatives. Except for age at menarche, all data were updated on subsequent questionnaires.

### Statistical analyses

Women contributed person-years from the beginning of follow-up in March 1995 until diagnosis of breast or other cancer, death, or end of follow-up in March 2017, whichever occurred first (970,189 total person-years). We used Cox proportional hazards regression models, stratified by age in 1-year intervals and questionnaire cycle such that age was the underlying time scale, to estimate hazard ratios (HRs) and 95% confidence intervals (CIs) for use of aspirin in relation to risk of overall, ER+, ER−, and TN breast cancer, separately. Aspirin use was treated as time-varying and modeled as a categorical exposure variable (non-use, past use, current use); over follow-up, current users were re-classified as past users if they no longer reported current regular use on subsequent questionnaires. In secondary analyses, we applied a 2-year lag to exposure classification, starting follow-up in 1997 and assigning aspirin use for each cycle according to that reported on the previous questionnaire. We also considered the duration of use among current users (< 5 years, 5–10 years, or ≥ 10 years). Finally, we also evaluated possible associations with the use of acetaminophen, which is an analgesic but does not have anti-inflammatory properties.

Multivariable models included adjustment for established breast cancer risk factors and potential confounders: recent body mass index (BMI < 25, 25–29.9, 30–34.9, or ≥ 35 kg/m^2^), age at menarche (< 11 years, 11 years, 12–13 years, or ≥ 14 years), recency of oral contraceptive use (never, < 5 years ago, 5 to < 10 years ago, or ≥ 10 years ago), parity (nulliparous, 1 birth, 2 births, or ≥ 3 births), age at first birth (< 20 years, 20 to < 25 years, or ≥ 25 years), lactation (never or ever), menopausal status (premenopausal or postmenopausal), duration of estrogen plus progestin use (never, < 5 years, or ≥ 5 years), alcohol consumption (not current; current, 1–6 drinks/week; or current, ≥ 7 drinks/week), physical activity (none, < 5 h/week, or ≥ 5 h/week), and first-degree family history of breast cancer (yes or no). Additional models further adjusted for the current use of other NSAIDs (non-use, past use, or current use). We also evaluated potential confounding by recent mammography use. Results were virtually unchanged; therefore, mammography use was not retained in the final multivariable models. Time-varying risk factors were updated at each questionnaire cycle. Missing indicator categories were used to account for missing information in covariates (generally 2–4%). To test whether associations differed by ER status, we used the contrast test method for heterogeneity by subtype [14].

In sensitivity analyses, we considered a referent group of women who never reported regular use of aspirin or other NSAIDs. We also evaluated whether associations differed within strata defined by BMI (< 30 kg/m^2^ vs. ≥ 30 kg/m^2^), waist-to-hip ratio (< 0.85 vs. ≥ 0.85), menopausal status (premenopausal vs. postmenopausal), age (< 50 vs. ≥ 50 years), and parity/lactation (nulliparous vs. parous, breastfed vs. parous, never breastfed). All analyses were performed using SAS 9.4 (Cary, NC).

## Results

At baseline, current regular users of aspirin were older (mean age, 44 years) and heavier (mean BMI, 29.1 kg/m^2^) than non-users (mean age, 38 years; mean BMI, 27.7 kg/m^2^). Compared to non-users, current users were also more likely to consume alcohol (39% vs. 28%) and less likely to have attained more than a high school education (72% vs. 83%). Current aspirin users and non-users generally had similar distributions of reproductive factors (Table 1).

**Table 1:** Age-standardized baseline characteristics according to current aspirin use

	Current aspirin use	
	No (*n* = 47,591)	Yes (*n* = 5535)
Age, years*	37.8 (10.2)	43.9 (11.7)
Current BMI, kg/m^2^	27.7 (6.6)	29.1 (7.2)
First-degree family history of breast cancer	9	9
Postmenopausal, %	16	18
Age at menarche		
< 11 years, %	11	13
11 years, %	17	16
12–13 years, %	52	50
≥ 14 years, %	19	21
Recency of oral contraceptive use		
Never, %	23	26
< 5 years ago, %	28	24
5–10 years ago, %	11	11
≥ 10 years ago, %	38	39
Postmenopausal hormone use		
Never used estrogen plus progestin (E+P), %	96	96
Used E+P, < 5 years duration, %	3	3
Used E+P, ≥ 5 years duration, %	1	1
Parity		
Nulliparous, %	36	33
Parous, 1 birth, %	21	20
Parous, 2 births, %	23	22
Parous, ≥ 3 births, %	19	23
Age at first birth (among parous)		
< 20 years, %	33	41
20–25 years, %	35	35
≥ 25 years, %	30	21
Ever lactation (among parous), %	52	48
Alcohol consumption		
Not current drinker, %	71	60
Current, 1–6 drinks/week, %	22	29
Current, ≥ 7 drinks/week, %	6	10
Educational attainment		
≤ 12 years, %	17	27
13–15 years, %	36	39
≥ 16 years, %	47	33

Values are means (SD) or percentages and are standardized to the age distribution of the study population

*Value is not age adjusted

Age- and multivariable-adjusted models produced similar results (Table 2). The multivariable-adjusted HR for overall breast cancer risk associated with current regular use of aspirin compared to non-use was 0.92 (95% CI 0.81, 1.04). Corresponding HRs for ER− and ER+ breast cancer were 0.81 (95% CI 0.64, 1.04) and 0.97 (95% CI 0.83, 1.13), respectively; however, there was no statistically significant heterogeneity by ER status (*p* = 0.20). Reductions in risk of ER+ breast cancer were noted for current users who had used aspirin regularly for at least 10 years (HR 0.81; 95% CI 0.60, 1.09). For ER−, there were no apparent trends in association with longer duration. Current regular use of aspirin was inversely associated with TN breast cancer (HR 0.70; 95% CI 0.49, 0.99), while there was no apparent trend in the duration of use (Table 3). Past use of aspirin was not associated with breast cancer risk overall, or by subtype (Tables 2 and 3). Further adjustment for the use of other NSAIDs did not change results (data not shown). Results were similar when exposure was lagged by 2 years (Additional Table 1) and when comparing current regular aspirin users to non-users of any NSAIDs (Additional Table 2). Multivariable-adjusted HRs for the association of current regular acetaminophen use and overall breast cancer, ER+ breast cancer, and ER− breast cancer were 0.88 (95% CI 0.74, 1.05), 0.88 (95% CI 0.68, 1.12), and 0.95 (95% CI 0.69, 1.31), respectively.

**Table 2:** Multivariable-adjusted hazard ratios (95% CI) for associations of aspirin and acetaminophen use and risk of breast cancer in the BWHS, overall and by ER status, 1995–2017

		All invasive (***n*** = 1919)					ER-positive (***n*** = 1112)					ER-negative (***n*** = 569)				
	Person-years	Cases	HR^a^	95% CI	HR^b^	95% CI	Cases	HR^a^	95% CI	HR^b^	95% CI	Cases	HR^a^	95% CI	HR^b^	95% CI
**Aspirin use**																
Non-use	676,158	1226	1.00	Reference	1.00	Reference	662	1.00	Reference	1.00	Reference	385	1.00	Reference	1.00	Reference
Past use	143,880	325	0.95	0.83, 1.08	0.97	0.85, 1.10	207	0.97	0.83, 1.14	0.98	0.83, 1.16	92	0.88	0.69, 1.11	0.92	0.72, 1.17
Current use	150,151	368	0.89	0.79, 1.01	0.92	0.81, 1.04	243	0.97	0.83, 1.13	0.98	0.84, 1.15	92	0.77	0.61, 0.98	0.81	0.64, 1.04
Duration of current use																
Current, < 5 years	73,790	173	0.91	0.77, 1.07	0.93	0.79, 1.09	108	0.98	0.80, 1.21	1.00	0.81, 1.23	43	0.75	0.55, 1.04	0.79	0.57, 1.09
Current, 5–10 years	41,940	117	0.96	0.79, 1.17	0.98	0.80, 1.19	82	1.09	0.86, 1.38	1.09	0.86, 1.39	28	0.80	0.54, 1.19	0.85	0.57, 1.27
Current, ≥ 10 years	34,422	78	0.77	0.61, 0.91	0.81	0.63, 1.03	53	0.78	0.58, 1.05	0.81	0.60, 1.09	21	0.76	0.48, 1.21	0.82	0.51, 1.31
**Acetaminophen use**																
Non-use	681,495	1340	1.00	Reference	1.00	Reference	755	1.00	Reference	1.00	Reference	406	1.00	Reference	1.00	Reference
Past use	208,818	439	0.94	0.84, 1.05	0.96	0.86, 1.08	283	0.98	0.85, 1.12	0.99	0.86, 1.15	119	0.83	0.67, 1.02	0.87	0.71, 1.08
Current use	79,876	140	0.85	0.71, 1.02	0.88	0.74, 1.05	74	0.86	0.67, 1.09	0.88	0.68, 1.12	44	0.91	0.67, 1.25	0.95	0.69, 1.31

^a^Adjusted for age

^b^Adjusted for age, first-degree family history of breast cancer, menopausal status, recent BMI, duration of E+P use, parity, age at first birth, age at menarche, recency of OC use, alcohol consumption, physical activity, and lactation

**Table 3:** Multivariable-adjusted hazard ratios (95% CI) for associations of aspirin use and risk of triple-negative breast cancer in the BWHS, 1995–2017

Aspirin use		Triple negative (***n*** = 284)				
	Person-years	Cases	HR^a^	95% CI	HR^b^	95% CI
Non-use	674,961	178	1.00	Reference	1.00	Reference
Past use	143,603	61	0.98	0.72, 1.32	0.99	0.72, 1.35
Current use	149,804	45	0.66	0.47, 0.94	0.70	0.49, 0.99
Duration of current use						
Current, < 5 years	36,817	14	0.48	0.28, 0.84	0.50	0.29, 0.87
Current, 5–10 years	20,923	18	0.92	0.56, 1.51	0.98	0.59, 1.61
Current, ≥ 10 years	17,183	13	0.70	0.39, 1.27	0.75	0.41, 1.37

^a^Adjusted for age

^b^Adjusted for age, first-degree family history of breast cancer, menopausal status, recent BMI duration of E+P use, parity, age at first birth, age at menarche, recency of OC use, alcohol consumption, physical activity, and lactation

Table 4 presents results within strata of BMI, waist-to-hip ratio, menopausal status, age, and parity/lactation status for overall, ER+, and ER− breast cancer. Associations were similar across BMI categories, whereas inverse associations with current aspirin use were observed for both ER+ and ER− breast cancer among women with a high waist-to-hip ratio (≥ 0.85) but not among those with waist-to-hip ratio < 0.85. Associations were consistent across menopausal status. However, the associations observed in the overall data were present among women ≥ 50 years old [HRs 0.89 (0.77, 1.03), 0.96 (0.80, 1.15), and 0.76 (0.57, 1.01), for overall, ER+, and ER− breast cancer, respectively], while there were no apparent associations for women < 50 years of age. Finally, the inverse associations noted for ER− breast cancer overall were apparent among parous women, but not among nulliparous women: among parous women who had breastfed, the HR was 0.69 (95% CI 0.45, 1.05), and among parous women who had never breastfed, the HR was 0.84 (95% CI 0.59, 1.19).

**Table 4:** Multivariable-adjusted hazard ratios (95% CI) for associations of aspirin use and risk of breast cancer in the BWHS, overall and by ER status, stratified by selected risk factors, 1995–2017

	Person-years	Cases	HR	95% CI	Cases	HR	95% CI	Cases	HR	95% CI
		**All invasive (*n*** **= 1127)**			**ER-positive (*n*** **= 626)**			**ER-negative (*n*** **= 353)**		
BMI < 30 kg/m^2^										
Non-use	435,269	768	1.00	Reference	397	1.00	Reference	250	1.00	Reference
Past use	76,978	176	1.01	0.85, 1.21	113	1.10	0.87, 1.37	51	0.89	0.64, 1.23
Current use	77,740	183	0.92	0.77, 1.09	116	1.00	0.80, 1.25	52	0.84	0.61, 1.16
		**All invasive (*n*** **= 792)**			**ER-positive (*n*** **= 446)**			**ER-negative (*n*** **= 216)**		
BMI ≥ 30 kg/m^2^										
Non-use	240,890	458	1.00	Reference	265	1.00	Reference	135	1.00	Reference
Past use	66,902	149	0.93	0.77, 1.13	94	0.89	0.70, 1.14	41	0.97	0.67, 1.38
Current use	72,411	185	0.91	0.76, 1.09	127	0.96	0.76, 1.20	40	0.77	0.53, 1.12
		**All invasive (*n*** **= 947)**			**ER-positive (*n*** **= 532)**			**ER-negative (*n*** **= 288)**		
WHR < 0.85										
Non-use	358,866	635	1.00	Reference	336	1.00	Reference	201	1.00	Reference
Past use	60,791	136	0.98	0.80, 1.19	82	0.97	0.75, 1.25	41	0.91	0.64, 1.30
Current use	69,650	176	0.98	0.82, 1.17	114	1.07	0.85, 1.34	46	0.84	0.60, 1.19
		**All invasive (*n*** **= 459)**			**ER-positive (*n*** **= 259)**			**ER-negative (*n*** **= 146)**		
WHR ≥ 0.85										
Non-use	137,202	273	1.00	Reference	145	1.00	Reference	87	1.00	Reference
Past use	36,330	96	1.04	0.81, 1.33	60	1.00	0.73, 1.38	31	1.15	0.74, 1.77
Current use	40,529	90	0.71	0.55, 0.92	54	0.67	0.48, 0.93	28	0.81	0.51, 1.28
		**All invasive (*n*** **= 710)**			**ER-positive (*n*** **= 370)**			**ER-negative (*n*** **= 226)**		
Premenopausal										
Non-use	436,127	577	1.00	Reference	290	1.00	Reference	185	1.00	Reference
Past use	52,186	76	1.01	0.79, 1.29	46	1.05	0.76, 1.46	26	1.02	0.67, 1.56
Current use	42,230	57	0.84	0.64, 1.11	34	0.95	0.66, 1.36	15	0.67	0.39, 1.15
		**All invasive (*n*** **= 966)**			**ER-positive (*n*** **= 610)**			**ER-negative (*n*** **= 261)**		
Postmenopausal										
Non-use	164,576	481	1.00	Reference	285	1.00	Reference	139	1.00	Reference
Past use	71,966	212	0.97	0.82, 1.16	139	0.96	0.77, 1.19	55	0.95	0.68, 1.32
Current use	93,480	273	0.91	0.78, 1.06	186	0.95	0.78, 1.15	67	0.87	0.64, 1.18
		**All invasive (*n*** **= 766)**			**ER-positive (*n*** **= 385)**			**ER-negative (*n*** **= 249)**		
Age < 50										
Non-use	476,929	620	1.00	Reference	305	1.00	Reference	200	1.00	Reference
Past use	59,268	79	0.97	0.76, 1.24	44	0.96	0.69, 1.33	28	1.03	0.63, 1.55
Current use	45,174	67	0.98	0.76, 1.27	36	1.06	0.75, 1.51	21	0.95	0.60, 1.49
		**All invasive (*n*** **= 1153)**			**ER-positive (*n*** **= 727)**			**ER-negative (*n*** **= 320)**		
Age ≥ 50										
Non-use	199,229	606	1.00	Reference	357	1.00	Reference	185	1.00	Reference
Past use	84,612	246	0.96	0.82, 1.12	163	0.98	0.81, 1.20	64	0.85	0.63, 1.15
Current use	104,977	301	0.89	0.77, 1.03	207	0.96	0.80, 1.15	71	0.76	0.57, 1.01
		**All invasive (*n*** **= 405)**			**ER-positive (*n*** **= 246)**			**ER-negative (*n*** **= 103)**		
Nulliparous										
Non-use	207,423	275	1.00	Reference	155	1.00	Reference	75	1.00	Reference
Past use	33,671	70	1.14	0.86, 1.51	54	1.31	0.94, 1.82	11	0.76	0.40, 1.46
Current use	31,741	60	0.87	0.64, 1.18	37	0.83	0.57, 1.22	17	1.11	0.63, 1.95
		**All invasive (*n*** **= 659)**			**ER-positive (*n*** **= 388)**			**ER-negative (*n*** **= 202)**		
Parous, breastfed										
Non-use	250,056	427	1.00	Reference	232	1.00	Reference	141	1.00	Reference
Past use	62,547	108	0.97	0.77, 1.21	69	1.06	0.79, 1.41	31	0.82	0.54, 1.24
Current use	69,258	124	0.95	0.76, 1.18	87	1.18	0.90, 1.54	30	0.69	0.45, 1.05
		**All invasive (*n*** **= 850)**			**ER-positive (*n*** **= 475)**			**ER-negative (*n*** **= 262)**		
Parous, never breastfed										
Non-use	199,229	522	1.00	Reference	275	1.00	Reference	167	1.00	Reference
Past use	84,612	146	0.91	0.75, 1.11	83	0.79	0.61, 1.03	50	1.10	0.78, 1.55
Current use	104,977	182	0.91	0.76, 1.09	117	0.89	0.71, 1.13	45	0.84	0.59, 1.19

*BMI* body mass index, *WHR*, waist-to-hip ratio

HRs are adjusted for age, first-degree family history of breast cancer, menopausal status (except analyses stratified by menopausal status), recent BMI (continuous), duration of E+P use, parity (except analyses stratified by parity/lactation), age at first birth, age at menarche, recency of OC use, alcohol consumption, physical activity, and lactation (except analyses stratified by parity/lactation)

## Discussion

Previously, we reported a significant inverse association of current regular aspirin use with breast cancer in the BWHS (HR 0.79; 95% CI 0.66, 0.95) [11]. Results of the present analysis, updated with 10 additional years of follow-up and > 1900 incident invasive breast cancer cases in total, are in the same direction but weaker when considering breast cancer risk overall; however, they suggest that current regular aspirin use may be associated with a nearly 20% reduction in risk of ER− breast cancer and a 30% reduction in risk of TN breast cancer among African American women. These findings warrant confirmation in other studies that include large numbers of African American women.

A recent meta-analysis reported a small decreased risk of breast cancer associated with current regular aspirin use [relative risk (RR) 0.90; 95% CI 0.85, 0.95]; however, the association was primarily apparent among population-based case-control studies (RR 0.80; 95% CI 0.73, 0.88) and hospital-based case-control studies (RR 0.82; 95% CI 0.75, 0.91) with no association among cohort studies (RR 0.97; 95% CI 0.90, 1.04) or nested case-control studies (RR 0.91; 95% CI 0.82, 1.01) [15]. Epidemiologic studies that evaluated associations of aspirin use with ER+ and ER− breast cancer risk separately have had inconsistent findings. In the California Teachers Study (CTS), taking 3 or more tablets per week of low-dose aspirin (but not regular strength aspirin) was associated with a reduced risk of breast cancer overall, and especially for the ER+/HER2− subtype (HR 0.80; 95% CI 0.66, 0.96) with no association for ER− breast cancer [16]; however, a prior analysis within the CTS showed a statistically significant increased risk of ER− breast cancer associated with daily, long-term use of aspirin (unspecified dose) [17]. A 20% reduction in risk of breast cancer associated with regular aspirin use was reported among postmenopausal women in the Iowa Women’s Health Study; similar decreased risks were noted for both ER+ and ER− breast cancer [18]. Among postmenopausal women enrolled in the VITamins And Lifestyle Study, a reduced risk of breast cancer was only apparent for women who used low-dose aspirin at least 4 times a week over 10 years (HR 0.65; 95% CI 0.43, 0.97); however, the corresponding HR for regular or extra-strength aspirin was 1.43 (95% CI 1.02, 2.00) [19]. No differences were seen by ER status [19]. Duration of aspirin use was inversely associated with breast cancer risk among postmenopausal women in the Women’s Health Initiative Observational Study (RR for ≥ 10 years of use vs. none 0.79; 95% CI 0.60, 1.03) [20]. These results are similar to our findings of an approximately 20% reduction in overall breast cancer risk with ≥ 10 years of aspirin use. In the NIH-AAPR Study (primarily postmenopausal women), daily use of aspirin was associated with lower risk of ER+ breast cancer (RR 0.84; 95% CI 0.71, 0.98) whereas no association was noted for ER− disease [21]. In the Sister Study, a prospective cohort of women who had a sister with breast cancer, greater lifetime use of aspirin based on frequency and duration was associated with a significantly reduced risk of breast cancer among premenopausal women (but not among postmenopausal women); subtype-specific analyses were not carried out within strata defined by menopausal status [22].

There have also been large prospective studies that reported no association or a positive association between aspirin use and breast cancer risk. Among postmenopausal women in the Nurses’ Health Study (NHS) and among premenopausal women in the NHSII, there was no association between current regular aspirin use (at least 2 tablets per week) and breast cancer risk overall or by ER status [23–25]. Results were similarly null in the Multiethnic Cohort (MEC) [26], the Cancer Prevention Study II Nutrition Cohort [27], and the European Prospective Investigation into Cancer and Nutrition Cohort, which included 7379 breast cancer cases over a median of 13.1 years of follow-up [28]. Aspirin users in the Danish Diet, Cancer, and Health cohort, however, experienced a statistically significantly increased risk of breast cancer compared to non-users (RR 1.38; 95% CI 1.12, 1.69) [29]. Only a single randomized controlled trial has been published: the Women’s Health Study showed no association of low-dose aspirin every other day with risk of invasive breast cancer, overall or by subtype, over an average of 10 years of follow-up [30, 31]. Inconsistencies in results from prior epidemiologic studies may partly reflect differences in exposure assessment and/or classification by frequency, duration, dose, or timing.

Besides the BWHS, the MEC is the only other prospective cohort study to report on the relation of aspirin use to breast cancer risk among African American women (HR for ≥ 6 years of use vs. non-use 0.98; 95% CI 0.64, 1.52); stratification by ER status was not reported [26]. In the Carolina Breast Cancer Study, a population-based case-control study, inverse associations of NSAID use and breast cancer risk were apparent among both white and African American women, but somewhat stronger among African American women [odds ratio (OR) for any use vs. no use of NSAIDs was 0.3 (95% CI 0.2, 0.6) for African American women compared to 0.5 (95% CI 0.3, 0.8) for whites]; that study did not consider subtypes of breast cancer [32]. No other studies have reported on these associations among African American women specifically, nor have any evaluated possible effect measure modification by race.

Aspirin and other NSAIDs could modulate breast cancer risk via their anti-proliferative, anti-angiogenic, and pro-apoptotic properties. Aspirin inhibits cyclooxygenase (COX), which is necessary for prostaglandin synthesis. Low-dose aspirin is specific for platelet COX-1, while the proposed anti-cancer effects are hypothesized to operate primarily through the COX-2 pathway [33]. Prostaglandins produced by COX-2 activity mediate inflammation and also increase aromatase expression, which leads to increased estrogen levels [5–7]. COX-2 inhibitors, including regular strength aspirin, may therefore reduce breast cancer risk by decreasing local estrogen production. Aspirin may also weakly inhibit aromatase through COX-2-independent pathways [34]. These mechanisms of action may be most relevant to hormone-dependent breast cancers. Compelling evidence suggests that inflammation may be a critical driver of ER− breast cancer [35–37] while higher levels of inflammatory cytokines [38] and genetic variants in cytokine-related genes [39] have been observed in African American women compared to white women. Inverse associations of aspirin with ER− and TN disease may therefore be driven by its anti-inflammatory effects rather than hormone-dependent mechanisms [40, 41]. Indeed, our observation of stronger associations among women 50 years and older, those with higher central adiposity, and parous women, groups with higher levels of systemic inflammation [42–46], is supportive of this theory; however, we were not able to evaluate potential mechanisms directly in this study.

An important limitation of this analysis is the lack of information on aspirin dose and possible misclassification of exposure based on self-report. Because we asked about regular use of aspirin defined as use at least 3 days per week, we were unable to distinguish whether more frequent use (e.g., daily use) shows stronger associations with breast cancer. Also, we did not evaluate non-aspirin NSAIDs separately because we did not consistently query the use of non-aspirin NSAIDs in the BWHS until 2009; however, adjustment for the use of non-aspirin NSAIDs reported on all questionnaires via open-text responses did not change effect estimates. Aspirin is a commonly used NSAID and may be expected to have a stronger protective effect on breast cancer because of its irreversible binding to COX-2 compared to non-aspirin NSAIDs [47]. We lacked information on reasons for use of aspirin; however, the lack of association for acetaminophen, which has a different mechanism of action than that of NSAIDs but is often used interchangeably with NSAIDs, suggests that the association of ER− or TN breast cancer with aspirin use is not likely explained by reasons for use. Temporal trends in aspirin use would not be expected to influence results since statistical models were adjusted for calendar time.

Strengths of this study include its prospective design and more than 20 years of follow-up, including repeated assessment of aspirin use over time which allowed us to update exposure information at each questionnaire cycle. Detailed characterization of breast cancer risk factors within the BWHS enabled careful control for potential confounding factors in our analyses. Finally, this is the largest study of aspirin use and breast cancer risk among African American women to date and we had a sufficient sample size to evaluate associations for subtypes of breast cancer defined by ER and HER2 status.

## Conclusions

The results of this study support the hypothesis that regular aspirin use is associated with reduced breast cancer risk, particularly for ER− and TN breast cancer, in African American women. If findings from this study are confirmed, aspirin may represent a potential opportunity for chemoprevention of ER− and TN breast cancer.

## Supplementary information

**Additional file 1 : Table S1.** Multivariable-adjusted hazard ratios (95% CI) for associations of lagged aspirin use in the BWHS, overall and by ER status, 1997-2017. **Table S2.** Multivariable-adjusted hazard ratios (95% CI) for associations of current regular aspirin use relative to non-use of any NSAIDs in the BWHS, overall and by ER status, 19952017.

## Data Availability

The datasets analyzed during the current study are available from the corresponding author on reasonable request.
